# Osteohistology of two phorusrhacids reveals uninterrupted growth strategy

**DOI:** 10.1002/ar.70135

**Published:** 2026-01-19

**Authors:** Lotta Dreyer, Christian Cooper, Jingmai O'Connor

**Affiliations:** ^1^ University of Hamburg Hamburg Germany; ^2^ Leibniz Institute for the Analysis of Biodiversity Change Hamburg Germany; ^3^ Negaunee Integrative Research Center Field Museum of Natural History Chicago Illinois USA

**Keywords:** Aves, histology, Oligocene, paleontology, Phorusrhacidae, South America

## Abstract

Phorusrhacidae were apex predators that primarily dominated South America ecosystems for at least 40 million years with their imposing size and predatory lifestyle—yet some aspects of their biology remain poorly understood. Osteohistology is a tool for understanding growth dynamics and biomechanical adaptations. Despite their ecological significance, histological analyses of these birds were non‐existent until the recent publication of a sampled metatarsus. Here, we present the first osteohistological data of a phorusrhacid femur together with other hindlimb elements representing two taxa, *Andrewsornis abbotti* and *Physornis fortis*, from the late Oligocene of Argentina. Ground‐section microscopy reveals highly vascularized fibrolamellar bone tissue, indicating rapid, sustained growth with lines of arrested growth appearing only in the outer circumferential layer. Extensive secondary remodeling, particularly at muscle attachment sites, highlights high mechanical stress, consistent with active cursorial locomotion. Body mass estimates (60–89 kg for *Andrewsornis abbotti*) and high relative cortical thickness in both specimens (bone compactness = 0.55–0.78) provide additional data for comparative studies. Our findings confirm the uninterrupted growth strategy of phorusrhacids, consistent with other large‐bodied flightless neognaths but in contrast to most giant flightless palaeognath birds.

## 1 INTRODUCTION

Phorusrhacidae, commonly referred to as “terror birds,” were a diverse family of large, predominantly flightless predatory birds that thrived throughout South America for much of the Cenozoic era before migrating to North America during the Pliocene (MacFadden et al., 2007). With fossil evidence ranging from the Eocene to the Late Pleistocene (Acosta Hospitaleche & Jones, 2024; Jones et al., 2018), these formidable neornithines occupied the apex predator niche, with estimated heights ranging from 80 cm up to 280 cm (Alvarenga & Höfling, 2003; Tambussi & Degrange, 2012). Despite their striking anatomical features and dominance in Paleogene and Neogene ecosystems, significant gaps remain in our understanding of their physiology, growth dynamics, and life history. While more extensive research has been conducted on phorusrhacid taxonomy, biomechanics, and paleoecology (Acosta Hospitaleche & Jones, 2024; Agnolín et al., 2025; Alvarenga & Höfling, 2003; Degrange, 2012; Degrange et al., 2010; LaBarge et al., 2024; Mayr, 2009), osteohistological data for this clade remain extremely limited. To date, only the poorly preserved tarsometatarsus of one phorusrhacid (*Patagornis marshi*) has been studied (Marsà et al., 2025). Notably, the identification of this specimen is tentative, given its poor preservation and the lack of geological data.

Osteohistology has proven to be a powerful tool for reconstructing the biology of extinct vertebrates, offering insights into growth patterns, metabolic rates, and skeletal adaptations (Chinsamy, 2023; De Buffrénil et al., 2021). Crown birds (Neornithes) are characterized by a specialized growth strategy in which adult size is achieved through uninterrupted growth within a year (Canoville et al., 2022; Padian & Horner, 2004). Growth lines, if present, are limited to the outermost cortex, the outer circumferential layer (OCL). This strategy evolved among non‐ornithurine ornithuromorphs during the Early Cretaceous and was inherited by crown birds (O'Connor et al., 2015). It has recently been observed that some flightless palaeognaths birds have secondarily evolved a protracted growth strategy, with bone deposition being prolonged over 1 year with growth lines interrupting bone tissue mid‐cortex. Although this was first observed in extinct giant flightless birds like moas (Dinornithiformes) and elephant birds (Aepyornithidae; Chinsamy, Angst, et al., 2020; Canoville et al., 2022), this pattern is also found in extant flightless taxa, such as ostriches (Struthioniformes), rheas (Rheiformes), emus (Dromaiidae), kiwis (Apterygidae), and cassowaries (Casuariidae; e.g., Bourdon et al., 2009; Heck & Woodward, 2021; Ong et al., 2022). Surprisingly, extinct giant flightless neognath taxa, such as *Brontornis*, *Gastornis*, and Dromornithidae, retain the rapid and mainly uninterrupted neornithine growth strategy (e.g., Canoville et al., 2022; Marsà et al., 2025); however, these groups have been sampled only rarely. These findings highlight the potential variability in growth dynamics among giant terrestrial birds and underscore the need for similar investigations in phorusrhacids. Given their unique evolutionary position within Neognathae and the disputed taxonomic position of other extinct giant neognaths (e.g., Agnolín, 2021; Worthy et al., 2017), phorusrhacid osteohistology represents a largely untapped source of information for reconstructing their biology.

Due to the destructive nature of thin sectioning, the relatively sparse fossil record of well‐preserved phorusrhacid fossils makes each histological sample a valuable resource. This study focuses on the histological analysis of long bone elements from two phorusrhacid specimens: *Andrewsornis abbotti* (FMNH P 14678; Patterson, 1941; Alvarenga & Höfling, 2003), represented by a complete femur, and *Physornis fortis* (FMNH P 13619; Ameghino, 1895; Alvarenga & Höfling, 2003), represented by fragments of a femur, tibiotarsus, and a tarsometatarsus. The specimens originate from the late Oligocene Cabeza Blanca locality in the Chubut province, Argentina, a fossil‐rich unit that has yielded a diverse array of vertebrates (Vucetich et al., 2015). *Andrewsornis abbotti* is the largest known member of the subfamily Patagornithinae and a mid‐sized phorusrhacid, while *Physornis fortis* purportedly represents one of the tallest phorusrhacids (Alvarenga & Höfling, 2003). After *Brontornis* was removed from Phorusrhacidae, the subfamily including *Physornis fortis* was renamed Physornithinae (Agnolín, 2007). Both taxa are only fragmentarily preserved in the fossil record and have not yet been the subject of histological analyses.

By conducting ground‐section microscopy, we aim to examine growth‐related structures, vascularization patterns, and remodeling processes within the bone tissue. Secondary osteon distribution may also offer insights into mechanical stress adaptations, providing valuable data on locomotion and potential cursorial constraints (Houssaye et al., 2016). Furthermore, the specimens differ in size and subfamily affiliation, enabling preliminary comparison of growth patterns across phorusrhacids under equivalent environmental and taphonomic conditions.

## 2 MATERIALS AND METHODS

### 2.1 Material

The fossils described in this paper are permanently housed in the Fossil Bird Collection of the Field Museum of Natural History (FMNH), Chicago (United States of America). All samples derive from Late Oligocene (Deseadan) deposits in the Chubut province in Argentina. The specimens were originally labeled as coming from the Deseado Formation (Loomis, 1914), a term no longer in use in modern stratigraphy. Based on more recent work (Carlini et al., 2021), the specimens can be confidently assigned to the Cabeza Blanca locality, which is part of the Sarmiento Formation. The formation is well‐known for its Oligocene fossil assemblages, including at least three different taxa of Phorusrhacidae (Carlini et al., 2021).

Both specimens represent distinct subfamilies within Phorusrhacidae and differ markedly in size and inferred locomotor adaptations, allowing for preliminary assessment of potential histological variation across the clade. Moreover, both specimens originate from the same locality, allowing their bone microstructure to be compared under equivalent environmental and taphonomic conditions.

*Andrewsornis abbotti* (FMNH P 14678) is preserved as an almost complete left femur (Figure 1a), missing only the medial condyle. It has a total length of 204 mm and a minimum circumference of 73 mm at the mid‐diaphysis.

**FIGURE 1:**
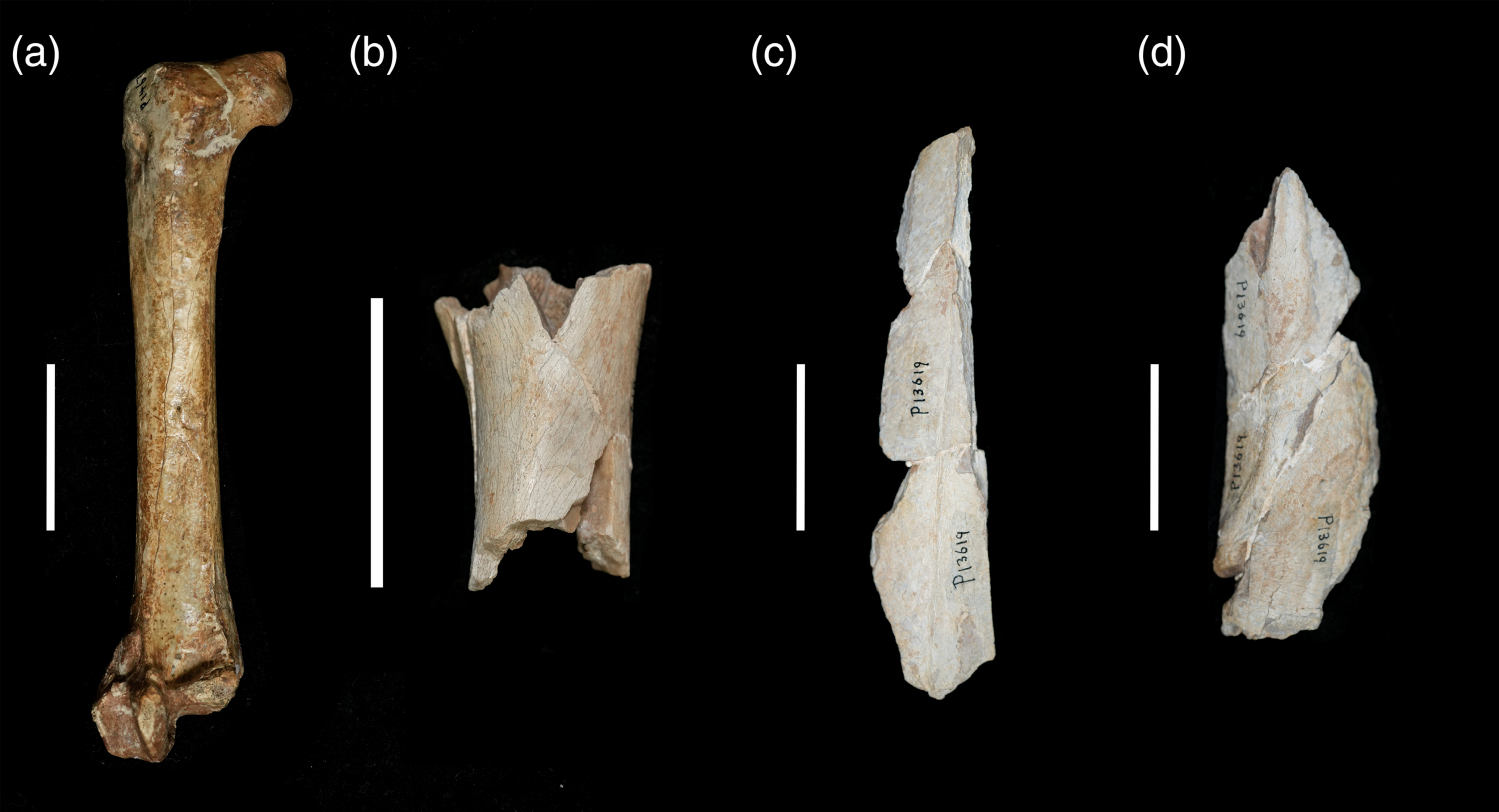
Phorusrhacid long bones sampled in this study. (a) Left femur of *Andrewsornis abbotti* (FMNH P 14678). (b) Tarsometatarsus fragment of *Physornis fortis* (FMNH P 13619). (c) Tibiotarsus fragment of *Physornis fortis* (FMNH P 13619). (d) Femur fragment of *Physornis fortis* (FMNH P 13619). Scale bars = 5 cm.

Three long bone elements from the same individual of *Physornis fortis* (FMNH P 13619) were sampled (Figure 1b–d). One fragment (Figure 1b) was initially identified as a humeral fragment based on general proportions. However, comparative size, morphology, and histological features suggest its reinterpretation as the metaphysis region of a tarsometatarsus. The identified femur (Figure 1d) consists of a curved shaft bearing a distinct longitudinal ridge. Our morphological analysis supports the original identification. Key diagnostic features include pronounced shaft curvature, a prominent intermuscular line (*linea intermuscularis*), and a robust, slightly stockier morphology compared to the tibiotarsus. The tibiotarsal fragment (Figure 1c) consists of a straight shaft with an oval cross‐section and a bony ridge, identified as the *facies articularis fibularis*. Therefore, *Physornis fortis* is represented by three hind limb elements.

### 2.2 Methods

We aimed to sample mid‐diaphyseal cross‐sections of the long bone elements, as is preferred for osteohistological analysis. Long bones are particularly suitable for histological assessment in vertebrate paleontology due to their appositional growth, complete cortical bone structure, and ability to record ontogenetic changes throughout an individual's lifespan (Chinsamy‐Turan, 2005). The mid‐diaphysis is considered the standard sampling location because it experiences minimal secondary remodeling compared to proximal or distal regions, providing a clearer record of primary bone deposition and vascularization patterns (Klein & Sander, 2008). This standardization allows for direct comparisons across taxa, facilitating broader interpretations of growth strategies and life history traits in extinct vertebrates. Due to the fragmentary nature of the *Physornis fortis* elements, their exact sampling location along the diaphysis could not be determined with precision. While the total lengths of the femur and tibiotarsus are unknown, the sampled fragments clearly correspond to the shaft region, as indicated by their general morphology and the absence of epiphyseal flaring in the associated osteological figures. In contrast, the reidentified tarsometatarsus section derives from the proximal metaphyseal region, limiting its comparative histological information.

The specimens were processed into ground sections following standard histological techniques (Chinsamy & Raath, 1992; Stein et al., 2009). Prior to sampling, all bones were photographed using a Sony α 7R digital camera. The femur FMNH P 14678 was molded and cast to preserve its external morphology before sectioning, whereas this was unnecessary for the femur, tibiotarsus, and tarsometatarsus fragments of FMNH P 13619. For FMNH P 14678, a small segment of the midshaft was removed using the Well® Diamond Wire Saw 6500. The segment was molded and recast to maintain completeness of the original femur. All samples (the removed segment of FMNH P 14678, respectively the fragments of FMNH P 13619) were embedded in polyester resin (Castolite® EP4117 Polyester Isophthalic Laminating Resin) that had been degassed in a vacuum chamber to minimize air bubbles which could interfere with microscopic observation. After curing, the embedded samples were sectioned transversely using the Buehler® IsoMet Low Speed saw. The cut surfaces were ground with Silicon Carbide grinding papers of the grit sizes 600 and 800 until smooth and free of saw marks. Each polished surface was then glued to a frosted glass slide using ARON ALPHA® Type 201 adhesive and left to harden completely. Once the adhesive was cured, the mounted blocks were cut to a thickness of a few millimeters using the manual saw on the Hillquist® Thin Section Machine and ground manually using the grinding wheel on the same device until optimal thickness was achieved. The ground sections were then manually ground again using finer Silicon Carbide grinding papers with grit sizes 800 and 1200. As a last step, the ground sections were polished with polishing paper using Buehler® MicroPolish Powder, 0.3 μm.

The finished ground sections were analyzed under an Olympus BX60 microscope and photographed using the Jenoptik GRYPHAX 2.1.0.724 system. Measurements were carried out in ImageJ version 2.16.0.

Bone compactness was calculated using BoneProfileR 3.1. Body mass was estimated using the equations from Campbell and Marcus (1992), table 2). Given the skeletal morphology of phorusrhacids, both the heavy‐bodied (HB: body mass (kg) = 10^0.1+2.131*LOG(femoral circumference (cm))^) and long‐legged (LL: body mass (kg) = 10^−0.216+2.316*LOG(femoral circumference (cm))^) bird equations were applied on FMNH P 14678 using its midshaft circumference of 7.3 cm. For the histological description, the presence and developmental degree of several histological features were analyzed, such as the Inner Circumferential Layer (ICL), Outer Circumferential Layer (OCL), and Lines of Arrested Growth (LAGs). Histological terminology follows Francillon‐Vieillot et al. (1990).

## 3 RESULTS

### 3.1 *Andrewsornis abbotti* (FMNH P 14678)

The histological cross‐section was taken from the midshaft of the femur. The cortical bone varies in thickness, ranging from 4.3 to 5.15 mm. The bone tissue consists of three distinct layers, an ICL, the primary bone tissue, and an OCL. Cortical bone is predominantly composed of reticularly organized vascular canals (Figure 2b). The composition and texture of the primary bone tissue show subtle changes throughout the cortex. The inner cortex is characterized by highly vascularized fibrolamellar bone (Figure 2b), suggesting intense osteogenesis during early growth stages. Moving outward, the bone organization transitions into less vascularized fibrolamellar bone, where vascular canals become more longitudinally oriented and osteocyte lacunae exhibit greater alignment parallel to the periosteal surface (Figure 2b). The cortical bone lacks cyclical growth marks such as LAGs or annuli, indicating uninterrupted bone formation throughout the individual's life.

**FIGURE 2:**
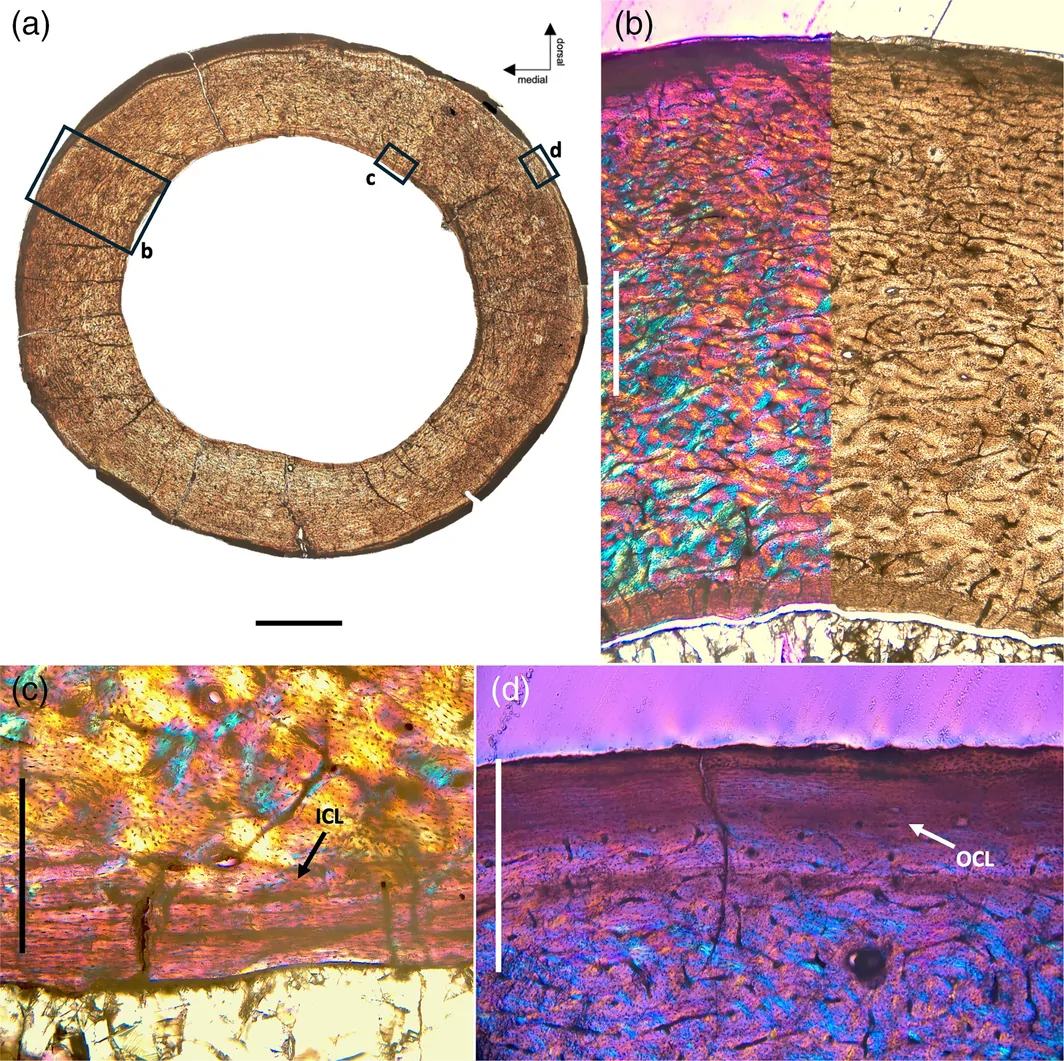
Femur of *Andrewsornis abbotti* (FMNH P 14678). (a) Overview of transverse section, with insert boxes showing the locations of (b)–(d). Scale bar = 5 mm. (b) Closeup from (a) showing the whole growth record in cross‐polarized (left) and normal (right) lights, revealing a plexiform vascularized fibrolamellar bone in the inner and more laminar organized fibrolamellar bone in the outer cortex. Scale bar = 1 mm. (c) Closeup from (a), showing the Inner Circumferential Line (ICL) in cross‐polarized lights. Scale bar = 250 μm. (d) Closeup from (a) of the Outer Circumferential Line (OCL) in cross‐polarized lights. Scale bar = 500 μm.

The presence of a distinct ICL (Figure 2c) marks the completion of medullary cavity expansion, while the appearance of an OCL (Figure 2d) indicates a substantial slowdown or even cessation of growth. The ICL forms a thin, circumferential band of parallel‐fibered bone along the medullary cavity, with an average thickness of 220 μm. The OCL, located directly beneath the periosteal surface, measures up to 300 μm in thickness, though it is partially eroded in some areas. Composed of parallel‐fibered bone as well, the OCL exhibits a marked reduction in vascular density compared to the underlying cortical tissue. Under normal light microscopy, this layer appears as a compact, darkened zone, and its organized nature is confirmed under polarized light. While OCLs often display closely spaced growth marks in other taxa (Ponton et al., 2004), LAGs are not discernible in this specimen due to the darkness of the OCL, and the occasional brighter patches also lack any discernible LAGs. Evidence of remodeling is limited, with only a few solitary secondary osteons scattered throughout the middle and outer cortex. Resorption cavities are absent.

The global bone compactness of FMNH P 14678 is 0.55. Depending on the applied equation from Campbell and Marcus (1992), FMNH P 14678 has an estimated body mass of 60.74 kg (LL), respectively 89.07 kg (HB).

### 3.2 *Physornis fortis* (FMNH P 13619)

#### 3.2.1 Femur

The femoral fragment of *Physornis fortis* (FMNH P 13619) shows a robust cortex with a cortical thickness between 10.1 and 14.89 mm. The cortical bone is dominated by disorganized, highly vascularized fibrolamellar bone (Figure 3a), interspersed with secondary osteons. Progressing towards the outer cortex, the organization of vascularization increases, transitioning from a reticular to laminar vascularization (Figure 3c). Both the ICL (Figure 3b) and OCL (Figure 3c) are present, with the ICL measuring 560 μm and the OCL 252 μm in average diameter. While no LAGs or other cyclical growth marks were observed in the primary cortical bone, there appears to be one in the OCL, though it could not be confirmed around the whole periosteal surface, as the OCL appears very dark and thin.

**FIGURE 3:**
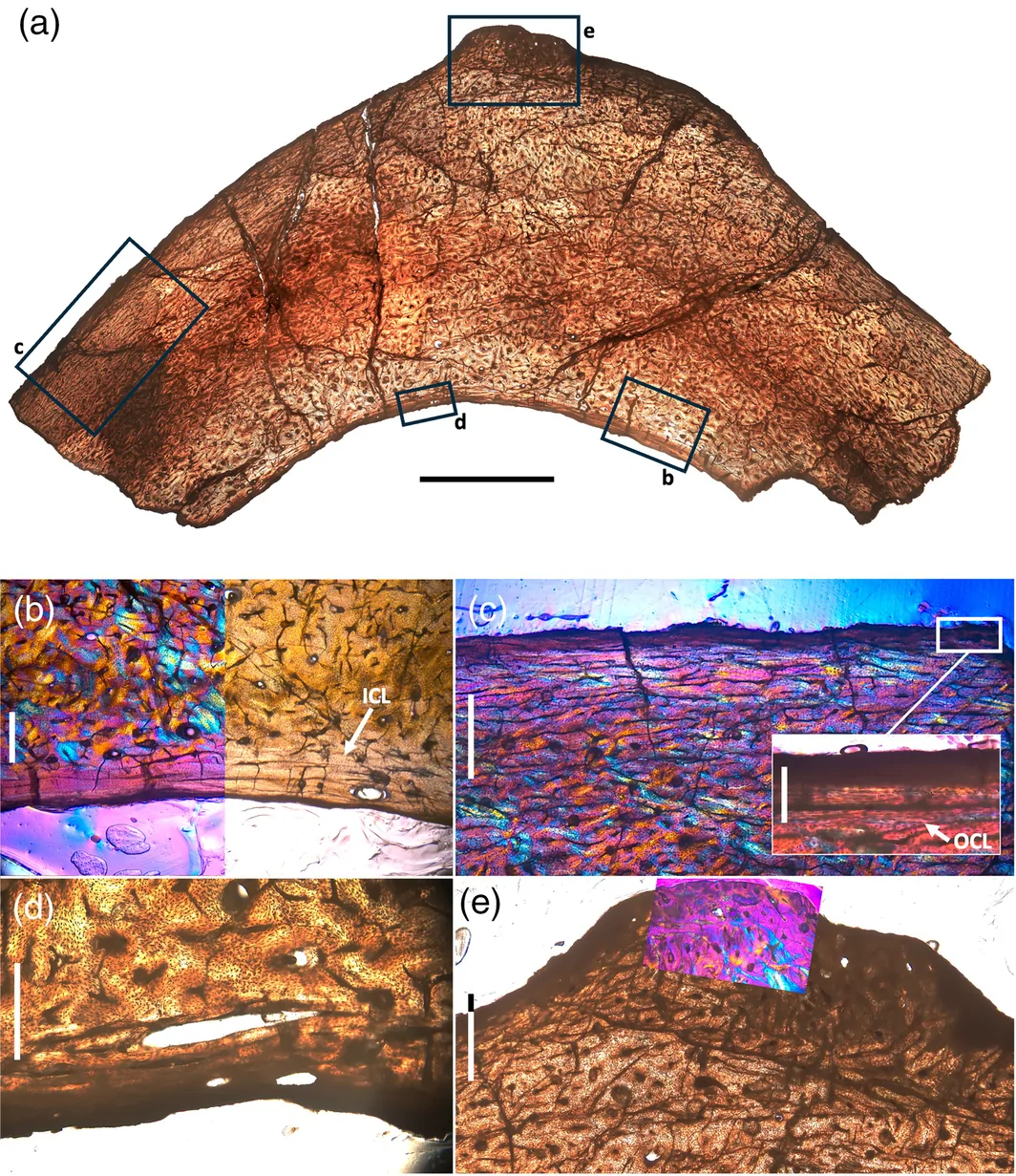
Femur of *Physornis fortis* (FMNH P 13619). (a) Overview of transverse section, with insert boxes showing the locations of (b)–(e). Scale bar = 5 mm. (b) Closeup from (a) showing the Inner Circumferential Line (ICL) in cross‐polarized (left) and normal (right) lights, revealing a plexiform vascularized fibrolamellar bone with a high abundance of secondary osteons in the inner cortex. Scale bar = 200 μm. (c) Closeup from (a), showing the outer cortex in cross‐polarized lights. The white box shows a closeup of the Outer Circumferential Line (OCL). Scale bar = 1 mm, respectively 250 μm for the inserted box. (d) Closeup from (a) of some smaller resorption cavities close to the endosteal surface under normal lights. Scale bar = 500 μm. (e) Closeup from (a), showing the highly remodeled *linea intermuscularis*, in both normal and cross‐polarized lights. Scale bar = 1 mm.

Small resorption cavities are present at the endosteal surface (Figure 3d). The inner and middle cortex are characterized by a higher density of secondary osteons (Figure 3b), reflecting extensive remodeling during later stages of life. A highly remodeled, longitudinal protuberance visible on the periosteal surface of the femur fragment corresponds to the *linea intermuscularis*, a prominent ridge along the midshaft that serves as the origin for the *m. femorotibialis* group (Baumel & Witmer, 1993; Degrange, 2012) (Figure 3e). Its presence further confirms that this fragment represents the broader diaphyseal region rather than the proximal or distal ends. This ridge is densely permeated by secondary osteons.

#### 3.2.2 Tibiotarsus

The tibiotarsus of *Physornis fortis* (FMNH P 13619) has a cortical diameter ranging from 10.82 to 11.24 mm. The cortex can be divided into ICL, partly remodeled primary bone, and OCL. The ICL measures approximately 266 μm in thickness, and the OCL about 222 μm. The ICL appears locally incomplete, but comparison with photographs of the specimen prior to sampling indicates that this discontinuity results from the fragmentary preservation of the endosteal surface and not a biological feature.

The primary bone tissue displays a vascular transition from disorganized, reticular fibrolamellar bone to more organized lamellar tissue towards the periosteal surface (Figure 4b,c). The inner cortex displays large resorption cavities (Figure 4b), indicative of extensive remodeling activity. Secondary osteons are distributed throughout the inner and middle cortex (Figure 4b,d). A small surface protuberance at the periosteal surface likely corresponds to the *linea extensoria*. It exhibits moderate remodeling, reflecting localized mechanical stress. Two closely spaced lines in the OCL (Figure 4e) likely represent LAGs, indicating a slowdown or cessation in growth during late ontogeny. No such lines were detected in the primary bone tissue.

**FIGURE 4:**
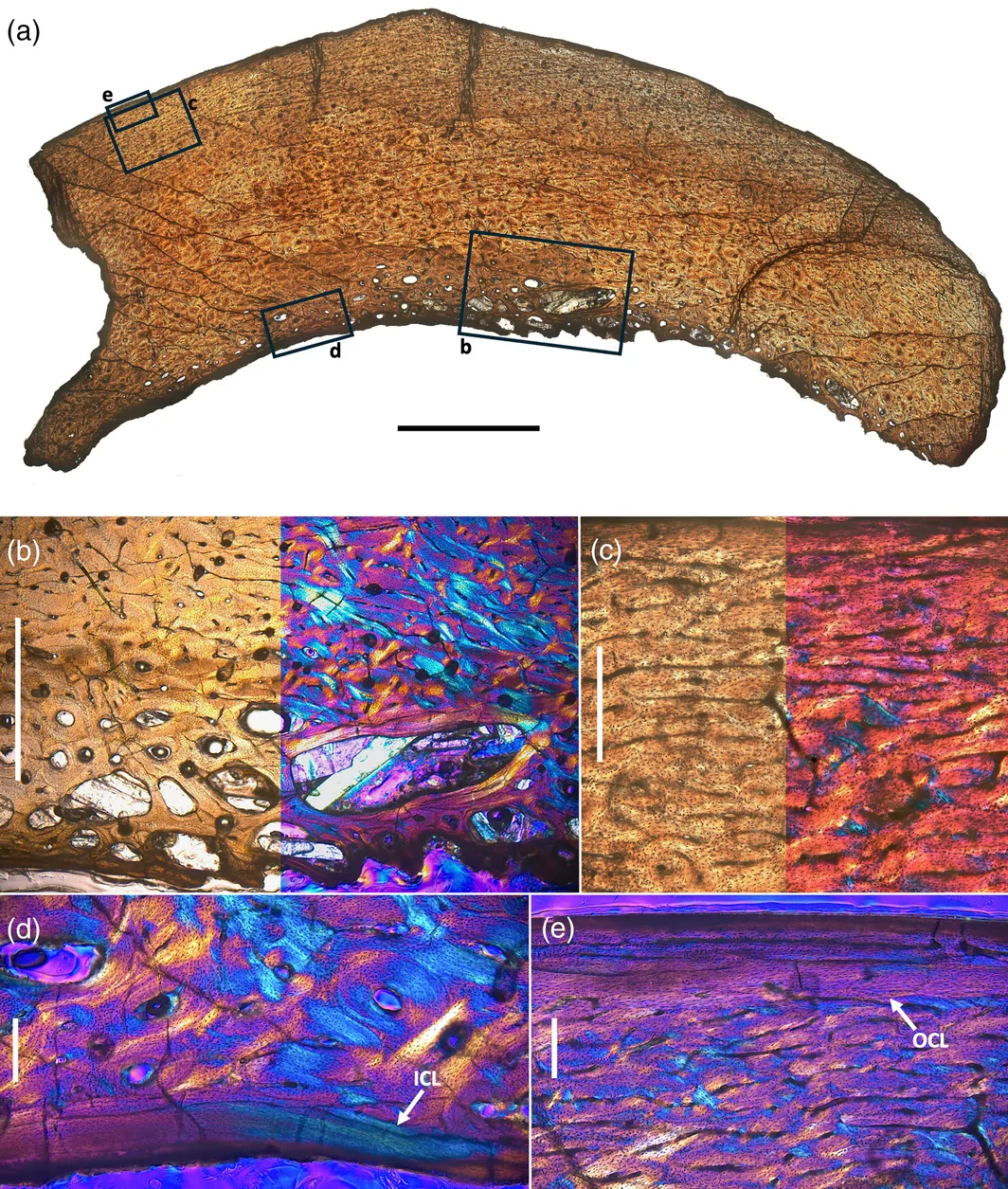
Tibiotarsus of *Physornis fortis* (FMNH P 13619). (a) Overview of transverse section, with insert boxes showing the location of (b)–(e). Curved lines traversing the mid‐ and outer cortex represent preparation artifacts and should not be interpreted as lines of arrested growth. Scale bar = 5 mm. (b) Inner cortex with plexiform vascularized, unorganized fibrolamellar bone and large resorption cavities in normal (left) and cross‐polarized (right) lights. Scale bar = 500 μm. (c) Outer cortex with a more laminar organization of vascular canals in normal (left) and cross‐polarized (right) lights. Scale bar = 500 μm. (d) Endosteal surface showing the Inner Circumferential Line (ICL) in cross‐polarized lights. Scale bar = 100 μm. (e) Periosteal surface showing the Outer Circumferential Line (OCL) in cross‐polarized lights. Scale bar = 100 μm.

#### 3.2.3 Tarsometatarsus

The cortical bone of the tarsometatarsus of *Physornis fortis* (FMNH P 13619) measures between 4.4 and 6.1 mm in thickness. The sub‐triangular cross‐section (Figure 5a) reveals extensive cortical remodeling, consistent with its metaphyseal origin. The ICL cannot be distinguished, likely due to intense remodeling of the endosteal region.

**FIGURE 5:**
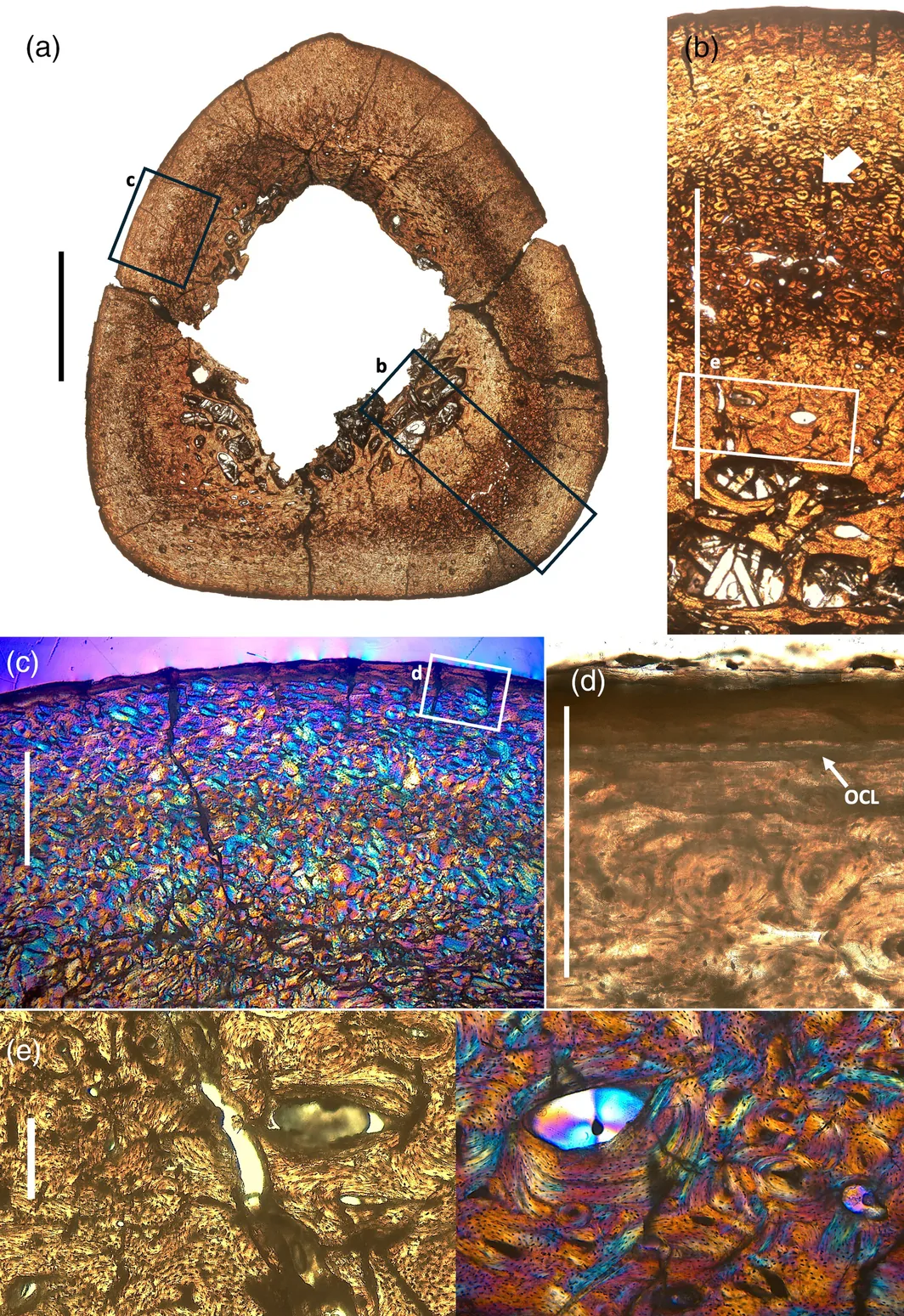
Tarsometatarsus of *Physornis fortis* (FMNH P 13619). (a) Overview of transverse section, with insert boxes showing the location of (b), (c), and (e). Scale bar = 5 mm. (b) Closeup from (a) showing the whole growth record in normal lights, revealing trabecular struts in the medullary cavity, large resorption cavities in the inner cortex and a thick band of haversian bone in the middle cortex (white arrow). Scale bar = 5 mm. (c) Outer cortex in cross‐polarized lights. No primary bone is visible due to the high density of secondary osteons obscuring the whole growth record. Scale bar = 500 μm. (d) Closeup from (c) showing the Outer Circumferential Layer (OCL), identifiable at only very few locations at the periosteal surface. Scale bar = 500 μm. (e) Closeup from (b) showing the inner cortex in normal (left) and cross‐polarized (right) lights. Resorption cavities are surrounded by bands of lamellar bone. Scale bar = 100 μm.

Most of the primary fibrolamellar bone has been replaced by secondary osteons (Figure 5b,c) and only very small patches of residual primary bone, consisting of woven, highly vascularized fibrolamellar bone, are visible in less remodeled regions. A conspicuous, darkly staining band of haversian bone is present in the mid‐cortex (Figure 5b). While it appears in the whole circumferential middle cortex, it is especially dense where flanked medially by trabecular struts extending into the medullary cavity (Figure 5b). Resorption cavities appear around the whole endosteal surface and are surrounded by a layer of lamellar bone (Figure 5e).

Cyclical growth marks are not visible within the primary bone due to remodeling. A thin OCL with one LAG is preserved at the periosteal surface (Figure 5d), however, it is obscured by secondary osteons at most places and appears very dark. A distinct *linea intermuscularis* is present on the cranial‐medial side of the femur.

Global compactness was calculated in this element as well; the result is 0.78.

## 4 DISCUSSION

### 4.1 Growth patterns in Phorusrhacids

The histological analysis of *Andrewsornis abbotti* (FMNH P 14678) and *Physornis fortis* (FMNH P 13619) reveals a consistent pattern of rapid growth, characterized by highly vascularized, plexiform fibrolamellar bone that transitions to more laminar and less vascularized tissue towards the outer cortex. Both specimens show no LAGs in the primary bone, suggesting a sustained, uninterrupted growth trajectory, with a reduction in growth rate only occurring later in ontogeny. Although the possibility that LAGs formed during early ontogeny were lost due to medullary expansion cannot be completely ruled out, this is very unlikely because the presence of rapidly deposited fibrolamellar bone together with an intact ICL indicates that medullary expansion has already ceased. Growth typically slows when approaching somatic maturity; therefore, if growth was periodic, LAGs should occur in later growth stages. An ICL and a thin OCL are present in all sampled elements, except for the tarsometatarsus of *Physornis*, where strong endosteal resorption and proximal sampling location obscure the ICL.

The OCL of *Andrewsornis abbotti* is too darkly mineralized to determine the presence or absence of LAGs in most places, and the few brighter patches of the OCL do not exhibit LAGs. In *Physornis fortis*, the OCL of the femur, tibiotarsus, and tarsometatarsus contains up to two closely spaced LAGs, indicating episodic late‐stage deposition after somatic maturity. Such a pattern is not unusual among extant or recently extinct birds, including the pied flycatcher (*Ficedula hypoleuca*; Dale, 2002) and the dodo (*Raphus cucullatus*; Angst et al., 2017). However, the extent of this post‐maturational growth cannot be determined with certainty, while LAGs restricted to the OCL indicate episodic growth, whether these marks represent annual events remains uncertain (Atterholt et al., 2025; Ponton et al., 2004).

The absence of LAGs in bright patches of the OCL in *Andrewsornis*, together with the lack of remodeling and resorption cavities and the absence of LAGs in the primary cortex may indicate a slightly earlier ontogenetic stage compared to *Physornis fortis*. Alternatively, the absence of visible LAGs could reflect individual or species‐level variation in growth patterns within Phorusrhacidae. Furthermore, less weight‐bearing stress could contribute to reduced remodeling in *Andrewsornis* (Cullen et al., 2020).

The initially misidentified humeral fragment of *Physornis fortis* (FMNH P 13619) is now recognized as a tarsometatarsus, based on morphological and histological evidence. Like the tarsometatarsus of *Patagornis marshi* (Marsà et al., 2025), the tarsometatarsal metaphysis fragment of *Physornis fortis* exhibits extensive secondary remodeling, with a dense Haversian system dominating the middle cortex. Overall, the tarsometatarsus shows high histological consistency with the associated femur and tibiotarsus, particularly where primary bone and the OCL are preserved. The remodeling is consistent with the trend towards greater remodeling observed in distal hind limb elements (De Ricqlès et al., 2016; Chinsamy & Worthy, 2021).

Although only three phorusrhacid individuals representing three species (including the previously sampled *Patagornis marshi*; Marsà et al., 2025) have been histologically studied to date, the addition of a completely sampled hind limb from *Physornis fortis* and a mid‐diaphyseal section from a complete femur of *Andrewsornis abbotti* provides important comparative data and the first opportunity to assess potential intraspecific and interspecific variation under comparable environmental conditions. Overall, the histological similarity between all three sampled taxa, despite their differences in overall body size, suggests a shared developmental strategy across phorusrhacids. Slight differences in LAG presence and remodeling might reflect variation in body size, ontogenetic stage, and allometric scaling effects associated with gigantism, potentially indicating subtle intraclade variation. The predominance of fibrolamellar bone and the absence of LAGs in primary bone supports the inference that uninterrupted growth was a common strategy within the group (Marsà et al., 2025).

### 4.2 Comparison in a phylogenetic context

The pattern found in *Andrewsornis abbotti* and *Physornis fortis* is typical in Neornithes, present in the earliest known members of the clade such as the volant Late Cretaceous *Vegavis* (Marsà et al., 2019) and in most extant members (Atterholt & Woodward, 2021; Dale, 2002; Williams, 2023).

Giant flightless taxa have evolved repeatedly in both palaeognaths and neognaths (Yonezawa et al., 2017). Among palaeognaths, most flightless taxa display a protracted, periodically interrupted growth strategy, with multiple LAGs distributed throughout the cortex (Table 1). This pattern is well documented in Aepyornithidae (Canoville et al., 2022; Chinsamy, Angst, et al., 2020), Dinornithidae (Canoville et al., 2022), Struthioniformes (Canoville et al., 2022; Ong et al., 2022), Casuariiformes, Rheiformes (Canoville et al., 2022), and Apterygiformes, including the small extant kiwi, which evolved from a large‐bodied ancestor (*Apteryx*; Bourdon et al., 2009). Exceptions exist (e.g., some specimens of *Dromaius* and *Rhea* exhibit only a few faint LAGs near the periosteal surface; Canoville et al., 2022; Ong et al., 2022), but the widespread occurrence of multiple well‐developed LAGs in the primary bone tissue in flightless palaeognaths indicates a reversal to cyclical growth—a pattern present in non‐ornithuromorph birds and inherited from non‐avian dinosaurs (Erickson et al., 2004; Atterholt et al., 2021). These cyclical growth patterns are consistent with the prolonged somatic maturity observed in most extant flightless palaeognaths, which typically require multiple years to reach adulthood (e.g., ostriches: 24–36 months; rheas: 20–24 months; kiwis: up to 6 years; Bourdon et al., 2009; Cooper et al., 2005; Sales, 2006).

**TABLE 1:** Comparison of locomotor and growth‐related histological characteristics between phorusrhacids and selected taxa, including extant and extinct birds and other archosaurs.

	Taxon	Locomotion	LAGs	OCL	Comp.	Reference
Neognathae	***Physornis fortis***	**Walking**	**Only in OCL**	**Yes**	**0.780**	This study and LaBarge et al. (2024)
	***Andrewsornis abbotti***	**Cursorial**	**No**	**Yes**	**0.553**	This study and LaBarge et al. (2024)
	*Patagornis marshi*	Cursorial	No	No	‐	Marsà et al. (2025) and LaBarge et al. (2024)
	*Brontornis burmeisteri*	Walking	No	No	‐	Agnolín (2021) and Marsà et al. (2025)
	*Gastornis* sp.	‐	Only in OCL	Yes	‐	Canoville et al. (2022)
	*Dromornithidae*	Walking	Only in OCL	Yes	‐	Chinsamy and Worthy (2021) and Chinsamy et al. (2023)
	*Raphus cucullatus*	‐	Only in OCL	Yes	‐	Brassey et al. (2016) and Angst et al. (2017)
	*Fregata aquila*	Volant	‐	‐	0.366	Fabbri et al. (2022)
Palaeognathae	Aepyornithidae	Walking	Yes	No	0.386–0.785	Chinsamy, Angst, et al. (2020) and Canoville et al. (2022)
	Dinornithidae	Walking	Yes	Yes	0.733–0.741	Canoville et al. (2022)
	*Apteryx*	Terrestrial	Yes	Yes	‐	Bourdon et al. (2009)
	*Struthio camelus*	Cursorial	Yes	Yes	0.289–0.702	Canoville et al. (2022) and Ong et al. (2022)
	*Dromaius novaehollandiae*	Cursorial	Yes	Yes	0.389–0.655	Canoville et al. (2022) and Ong et al. (2022)
	*Rhea americana*	Cursorial	Yes	Yes	0.404–0.826	Canoville et al. (2022)
	*Casuarius casuarius*	Cursorial	Yes	Yes	0.395	Canoville et al. (2022)
	*Eudromia*	Terrestrial, volant	Yes	No	0.282	Fabbri et al. (2022)
	Lithornithidae	Volant, swimming	No	‐	‐	Houde and Club (1988) and Widrig et al. (2025)
Enantiornithes	*Avimaia*	Arboreal/volant	Yes	No	0.458	Fabbri et al. (2022) and Atterholt et al. (2025)
Ichthyornithes	*Ichthyornis*	Volant	No	No	‐	Chinsamy & Marugán‐Lobón. (2020)
Hesperornithes	*Hesperornis*	Swimming/diving	No	Yes	0.725	Wilson (2019) and Fabbri et al. (2022)
Confuciusornithi‐dae	*Confuciusornis sanctus*	Volant/gliding	No	Yes	0.458	De Riqclès et al. (2003), Chinsamy, Marugán‐Lobón et al. (2020), Fabbri et al. (2022) and Chiappe et al. (2023)
Archosauria	*Allosaurus*	Graviportal	Yes	Yes (=EFS)	0.597	Fabbri et al. (2022)
	*Alligator*	Semi‐aquatic	Yes	Yes (=EFS)	0.884	Fabbri et al. (2022)

*Note*: Specimens from this study in bold.

Abbreviations: Comp., bone compactness after Gônet et al. (2021); EFS, external fundamental system; LAGs, lines of arrested growth, including only those in the primary bone tissue; OCL, outer circumferential line.

In contrast, all examined giant flightless neognaths retain rapid, sustained growth (Table 1). *Brontornis burmeisteri*, hypothesized to belong to Anseriformes (Agnolín, 2021), exhibits a dense fibrolamellar, well‐vascularized cortex with no growth marks (Marsà et al., 2025). The galloanseran *Gastornis* exhibits multiple closely spaced LAGs confined to the outermost primary bone tissue and OCL (Canoville et al., 2022), similar to the galloanseran dromornithids *Dromornis stirtoni* and *Genyornis newtoni* (Chinsamy & Worthy, 2021; Chinsamy et al., 2023). This histological signature corresponds with known patterns from extant neognaths (De Ricqlès et al., 2001). The new osteohistological data for phorusrhacids presented here does not only confirm the same fast growth strategy previously reported for *Patagornis marshi* (Marsà et al., 2025), but also strongly suggests that this pattern was retained in all giant flightless clades of neognaths.

It has yet to be fully understood why flightless neognath and palaeognath clades utilize different growth strategies to achieve gigantism. However, this phenomenon is not unknown: different clades of giant theropods also evolved large body sizes through unique growth strategies (D'Emic et al., 2023). In birds, uninterrupted growth evolved in the Late Cretaceous within a subset of Ornithuromorpha, the clade that includes Neornithes, being present in Early Cretaceous taxa such as *Iteravis* and *Yanornis* (O'Connor et al., 2015; Wang et al., 2020). Neognaths and palaeognaths are hypothesized to have diverged during the Late Cretaceous (LaBarge et al., 2024; Yonezawa et al., 2017). The oldest known fossil palaeognaths are the volant Paleocene Lithornithidae (Stidham et al., 2014; Widrig et al., 2025; Widrig & Field, 2022). Lithornithids display uninterrupted, rapid growth characterized by a transition from fibrolamellar to parallel‐fibered bone in the outer cortex (Houde & Club, 1988), indicating that at least early palaeognaths had the plesiomorphic rapid growth strategy characteristic of Neornithes. One of the earliest known palaeognath birds with protracted growth is the Eocene *Palaeotis* (Houde & Haubold, 1987), raising questions about when and why protracted growth was secondarily acquired. During the Paleogene, palaeognaths subsequently diversified in both insular and continental habitats, and gigantism evolved independently on multiple landmasses (Yonezawa et al., 2017).

Extreme gigantism in palaeognaths, such as *Aepyornis* (>500 kg; Hansford & Turvey, 2018), might have favored protracted growth. Yet this pattern is retained even in smaller flightless palaeognaths (e.g., *Casuarius*) and in the kiwi, a sister taxon of *Aepyornis* (Mitchell et al., 2014), indicating that body‐size reduction alone does not necessarily eliminate LAGs. Another suggested driver of protracted growth is insularity (e.g., Bourdon et al., 2009; Chinsamy, Angst, et al., 2020; Starck & Chinsamy, 2002; Turvey et al., 2005), although this claim is undermined by evidence that insular flightless neognaths like the dodo (Angst et al., 2017; Brassey et al., 2016) retain uninterrupted growth.

Our new data on phorusrhacids, together with a review of existing literature, suggest that growth strategy is most strongly determined by phylogeny. Legendre et al. (2014) quantitatively assessed the phylogenetic signal in avian osteohistology, showing that bone‐size‐related traits, including growth‐rate proxies, carry a strong phylogenetic component, further supporting the hypothesis that growth strategy is deeply constrained by lineage rather than by ecology alone. Rather than reverting to a protracted and cyclical growth strategy when evolving large body sizes, all studied giant flightless neognaths maintained uninterrupted growth and therefore reached adult body size more rapidly than giant palaeognaths. While insular conditions and body mass, as well as other ecological and environmental factors such as resource availability, seasonality, and predation (Chinsamy, 2023; Cooper et al., 2008; De Buffrénil & Quilhac, 2021), likely influence growth dynamics, the strong phylogenetic signal suggests that other drivers are acting within the limits imposed by deeply conserved, endogenous growth rhythms and phylogenetic developmental constraints. These intrinsic regulatory mechanisms appear to have constrained the evolutionary trajectories and growth patterns available to different avian lineages.

Future work integrating broader taxonomic sampling will be critical to fully understand how intrinsic developmental constraints interact with extrinsic pressures in shaping growth patterns across birds. Important histological data for continental giant palaeognaths (e.g., *Pachystruthio*; Zelenkov et al., 2019; Buffetaut & Angst, 2021) are lacking, as well as data for heavier phorusrhacids such as *Kelenken*. Data from these key taxa, together with a generally broader sampling matrix (Legendre et al., 2014), may allow a better understanding of growth‐pattern drivers in giant birds in the future.

### 4.3 Biomechanical implications

Remodeling progressing from the endosteal surface into the cortex is a marker of advancing ontogeny, molting, and metabolism. In contrast, concentrated secondary osteons in the outer cortex, like those observed in *Physornis fortis* (FMNH P 13619), are often associated with biomechanical demands (Angst et al., 2017; Chinsamy, Angst, et al., 2020).

Prominent longitudinal ridges are visible on the femur, tibiotarsus, and tarsometatarsus of *Physornis fortis*, representing *lineae intermusculares* (intermuscular lines) that mark muscle boundaries (Baumel & Witmer, 1993). Particularly extensive remodeling is evident in the femoral *linea intermuscularis*, a structure that defines the borders between the origins of the three parts of the *m. femorotibialis* (lateralis, intermedius, medialis; Degrange, 2012). The prominence of this *linea* and the remodeling within it indicate a well‐developed femoral musculature. Similarly, the *linea extensoria* on the tibiotarsus represents an origin site for the distal portions of the *m. femorotibialis* extensor muscles (Degrange, 2012), which are crucial for extending the femorotibial joint and generating the powerful propulsive strokes characteristic of cursorial locomotion in phorusrhacids. While the *Physornis* tarsometatarsus section is less informative for general growth patterns due to its proximity to the proximal end, its morphology and histological characteristics support biomechanical implications from the femur and tibiotarsus. The tarsometatarsus exhibits extensive secondary remodeling of the whole primary bone and a highly trabecular internal structure, and the presence of a distinct *linea intermuscularis* on the cranial‐medial surface marks the origin of the *m*. e*xtensor hallucis longus* (Degrange, 2012). The pronounced development of those lines in all three elements, together with extensive secondary remodeling at the periosteal surface and particularly within the *lineae*, large resorption cavities, and a highly trabecular internal structure in all hindlimb elements of *Physornis* suggest that these areas were sites of significant stress related to stance and propulsion. This histological data supports the hypothesis that *Physornis* possessed a robust hind‐limb musculature adapted for a heavy‐bodied bird that relied on powerful, relatively slow, sustained terrestrial walking rather than rapid running (Degrange, 2012; Houssaye et al., 2016; LaBarge et al., 2024).

Literature suggests that cursorial birds exhibit more biomechanically induced remodeling in periosteal regions than slow walkers, reflecting the high‐frequency, high‐impact loading associated with running (Canoville et al., 2022; Straehl et al., 2013). However, the femur of *Andrewsornis abbotti*, a cursorial phorusrhacid (LaBarge et al., 2024), shows little histological indication of periosteal mechanical adaptation. Although the *linea intermuscularis caudalis* is macroscopically discernible, it is only weakly developed in thin section, and only a few isolated secondary osteons are present in the inner cortex, with no significant trabecular differentiation. This limited, non‐directional remodeling suggests modest strain accommodation rather than focal mechanical reinforcement. While this does not contradict the interpretation of *Andrewsornis* as a predominantly cursorial taxon, the histological evidence itself remains biomechanically equivocal, and any further inference would be speculative.

The discrepancy in the degree of bone remodeling in the graviportal *Physornis* compared to the cursorial *Andrewsornis* can be reconciled by considering the overriding effect of absolute body mass on peak bone stresses. The greater body mass of *Physornis* would generate substantially higher ground‐reaction forces even during slow walking, thereby demanding more extensive secondary osteon formation for microdamage repair, alongside a relatively higher bone compactness. Ontogenetic differences, sampling location and sampled element, and phylogenetic baseline remodeling rates may also contribute to this pattern, as secondary osteon density tends to increase with ontogenetic age and varies among clades (Canoville et al., 2022; LaBarge et al., 2024). Additionally, cursorial and slow‐walking locomotion lie in a spectrum rather than representing a strict binary. While *Andrewsornis* likely occupied the faster end of this spectrum, it may not have experienced the extreme torsional and cyclic bending stresses typical of fully cursorial ratites (e.g., ostrich, emu). Conversely, *Physornis*, although traditionally described as “graviportal” or slow walking, could have performed intermittent, relatively rapid bouts of locomotion, such as short chases after prey, that produced peak stresses sufficient to trigger pronounced remodeling despite a generally slower gait (Angst et al., 2017).

### 4.4 Body mass and global bone compactness

Because *Andrewsornis* is only represented by a femur, body‐mass estimation must rely on single‐bone regressions, which are necessarily somewhat speculative for extinct taxa. We utilized the widely applied femoral circumference equations of Campbell and Marcus (1992), commonly used for large, flightless fossil birds (e.g., Hansford & Turvey, 2018; Chinsamy, Angst, et al., 2020; Canoville et al., 2022). This method has been critically assessed (Dickison, 2007) and shown to yield reliable correlation values and performance comparable to more recent models, useful when only a single robust hindlimb element is preserved.

Depending on the applied equation (long‐legged or heavy‐bodied; Campbell & Marcus, 1992), body mass estimates for *Andrewsornis abbotti* (FMNH P 14678) are between 60 and 89 kg. As a member of the subfamily Patagornithinae (Degrange et al., 2012), *Andrewsornis abbotti* is classified as a medium‐sized Phorusrhacidae (Degrange et al., 2010; LaBarge et al., 2024). Previously estimated phorusrhacid body masses range from 8 to 13 kg in small taxa like *Psilopterus lemoinei* (Degrange et al., 2012; Degrange & Tambussi, 2011) to 26–45 kg in medium‐sized taxa like *Patagornis marshi* (Alvarenga & Höfling, 2003; Degrange et al., 2012). Giant phorusrhacids, which include members of the Phorusrhacinae and Physornithinae (LaBarge et al., 2024), have been estimated as exceeding 100 kg, as in *Phorusrhacos longissimus* (Degrange et al., 2012; Degrange & Tambussi, 2011). With an estimate between 60 and 89 kg, *Andrewsornis abbotti* can be placed within the upper limit medium‐sized range for phorusrhacids. Interestingly, these estimates, even for the largest phorusrhacids, are well below the body masses of the largest palaeognath birds, like the extinct aepyornithids (107–642 kg; Hansford & Turvey, 2018; Hansford, 2018) and dinornithids (15–240 kg; Brassey et al., 2013) and even extant ostriches (90–150 kg; Deeming et al., 1996).

Global bone compactness (also referred to as bone density; see Fabbri et al., 2022) quantifies the relative size of the medullary cavity compared to the total bone cross section (Gônet et al., 2021), with higher values indicating a thicker cortex and lower values a thinner cortex. Volant birds generally have much lower compactness values than flightless taxa (e.g., *Eudromia*: 0.282; *Fregata*: 0.366). In a broader dataset of giant flightless birds, the *Andrewsornis* femur (0.553) falls in an intermediate range (see Table 1). In *Physornis fortis* (FMNH P 13619), compactness could only be measured in the tarsometatarsus, which shows a notably high value (0.780). However, this is less reliable due to the sampling location being near the proximal epiphysis. Comparative data from Canoville et al. (2022) showed that bone compactness increases along the hindlimb in a proximo‐distal direction, which may partly explain this high value.

Across all birds, bone compactness generally increases with body size (Table 1), but this trend becomes less apparent when focusing solely on giant flightless taxa (Canoville et al., 2022). Similarly, Aureliano et al. (2025) note that bone compactness does not scale predictably with body mass, reflecting a complex interplay of phylogenetic, biomechanical, and ecological factors. All giant flightless birds show intermediate to high femoral compactness values, for example, dinornithids (0.741) and aepyornithids (0.512), yet compactness can vary substantially within and between taxa. For instance, in Canoville et al. (2022), *Aepyornis* is represented by a larger femur than *Dinornis*, but the latter exhibits higher compactness. Within *Struthio camelus*, values range from 0.289 to 0.702, and differences cannot be entirely explained by ontogeny and only partly by the sampled element. No clear correlation exists between bone compactness and locomotor mode: both *Rhea americana* and aepyornithids can show values above 0.7 despite very different ecologies. These observations underscore that while general trends exist, bone compactness is influenced by multiple interacting factors and should not be interpreted solely as a function of body size or locomotion.

## 5 CONCLUSION

The histological analysis of *Andrewsornis abbotti* (FMNH P 14678) and *Physornis fortis* (FMNH P 13619) reveals a shared growth strategy characterized by rapid, uninterrupted bone deposition throughout most of ontogeny. Growth appears to decelerate only near skeletal maturity, as indicated by reduced vascularization and the presence of distinct OCLs. These findings indicate that phorusrhacids reached skeletal maturity fast and without interrupting growth, differing from the slower, episodic growth dynamics seen in other large flightless birds. The continuous growth pattern of phorusrhacids is consistent with other neognath lineages, including other large, flightless forms, and contrasts strongly with the interrupted growth typical of flightless palaeognaths. This difference indicates that each clade evolved unique developmental pathways to gigantism, with neognaths retaining uninterrupted growth and palaeognaths secondarily reverting to a protracted, cyclical strategy.

Although the sample size remains limited, this study constitutes a significant advance in understanding phorusrhacid growth strategies. It provides the first femoral osteohistological data, which permits reliable comparison with other sampled taxa, and includes histological evidence from multiple hindlimb elements in a single taxon. By encompassing individuals from two distinct phorusrhacid subfamilies, one of which is medium‐sized and the other one large, this work offers new insights into growth dynamics, skeletal maturity, and biomechanical adaptation. The results highlight the need for expanded osteohistological sampling across additional taxa and ontogenetic stages, forming a foundation for future comparative studies on the histology and life history evolution of extinct avian lineages.

## AUTHOR CONTRIBUTIONS

**Lotta Dreyer:** Writing – original draft; writing – review and editing; conceptualization; investigation; methodology; data curation; visualization. **Christian Cooper:** Formal analysis; software. **Jingmai O'Connor:** Supervision; writing – original draft; writing – review and editing; conceptualization; project administration; resources.

## FUNDING INFORMATION

This research received no specific grant from any funding agency in the public, commercial, or not‐for‐profit sectors.

## CONFLICT OF INTEREST STATEMENT

The authors declare no conflicts of interest.

## Data Availability

All data generated or analyzed during this study are included in this published article. Additional data are available from the corresponding author on reasonable request.
